# CRISPR/Cas9-mediated base-editing enables a chain reaction through sequential repair of sgRNA scaffold mutations

**DOI:** 10.1038/s41598-021-02986-6

**Published:** 2021-12-13

**Authors:** Tsuyoshi Fukushima, Yosuke Tanaka, Keito Adachi, Nanami Masuyama, Akiho Tsuchiya, Shuhei Asada, Soh Ishiguro, Hideto Mori, Motoaki Seki, Nozomu Yachie, Susumu Goyama, Toshio Kitamura

**Affiliations:** 1 Division of Cellular Therapy, The Institute of Medical Science, The University of Tokyo, Minato-ku, Tokyo, 108-8639 Japan; 2 Research Center for Advanced Science and Technology, The University of Tokyo, Tokyo, 153-8904 Japan; 3 School of Biomedical Engineering, The University of British Columbia, Vancouver, BC V6T 1Z3 Canada; 4 The Institute of Laboratory Animals, Tokyo Women’s Medical University, Tokyo, Japan; 5 Division of Molecular Oncology, Graduate School of Frontier Sciences, The Institute of Medical Science, The University of Tokyo, Minato-ku, Tokyo, 108-8639 Japan

**Keywords:** Biological techniques, Biotechnology

## Abstract

Cell behavior is controlled by complex gene regulatory networks. Although studies have uncovered diverse roles of individual genes, it has been challenging to record or control sequential genetic events in living cells. In this study, we designed two cellular chain reaction systems that enable sequential sgRNA activation in mammalian cells using a nickase Cas9 tethering of a cytosine nucleotide deaminase (nCas9-CDA). In these systems, thymidine (T)-to-cytosine (C) substitutions in the scaffold region of the sgRNA or the TATA box-containing loxP sequence (TATAloxP) are corrected by the nCas9-CDA, leading to activation of the next sgRNA. These reactions can occur multiple times, resulting in cellular chain reactions. As a proof of concept, we established a chain reaction by repairing sgRNA scaffold mutations in 293 T cells. Importantly, the results obtained in yeast or in vitro did not match those obtained in mammalian cells, suggesting that in vivo chain reactions need to be optimized in appropriate cellular contexts. Our system may lay the foundation for building cellular chain reaction systems that have a broad utility in the future biomedical research.

## Introduction

A chain reaction is a sequence of related events or reactions in which each event/reaction causes the next. Since many biological processes consist of sequential chemical or cellular reactions, methods and platforms that allow molecular recording or intervention of sequential events in living cells have broad applications in cell biology, bioengineering, and therapeutics. Several systems have been developed for recording chain reactions in mammalian cells. *Friedland *et al. developed two cellular chain reaction systems using T7/T3 RNA polymerases and Cre/Flippase (Flp) recombinases. In the first system, T7 RNA polymerase induced the expression of T3 RNA polymerase and T3 RNA polymerase triggered the next reaction^1^. In the second system, recombination of the flippase recognition target (FRT) by Flp induced Cre expression, and Cre-induced LoxP recombination triggered the next reaction. Since the number of RNA polymerases and recombinases is limited, the number of chain reactions in these systems is also limited. Recently, *Farzadfard *et al. developed an elegant chain reaction system called DOMINO^2^, using a nickase Cas9 tethering of a cytosine nucleotide deaminase (nCas9-CDA), resulting in efficient substitution of the cytosine nucleotide (C) with a thymidine nucleotide (T) in the genomic sequence^3^. In the DOMINO system, the C > T modification in the target DNA locus by sgRNA and nCas9-CDA triggered the next modification. *Farzadfard *et al. also achieved the conversion of inactive sgRNA to active sgRNA by introducing C > T substitutions into the protospacer sequence of nonfunctional sgRNA. One limitation of this system is the difficulty of generating multiple active sgRNAs during chain reactions because the protospacer sequence is strictly dependent on the sequence of target DNA.

To establish platforms that enable sequential activation of multiple sgRNAs, we developed two nCas9-CDA-based chain reaction systems by sequential repair of mutations in sgRNA scaffolds or TATAloxP sequences. We generated PAM-inserted sgRNAs without loss of function and successfully inactivated them by introducing T > C mutations that can be repaired by nCas9-CDA. As a proof of concept, we established a cellular chain reaction by repairing sgRNA scaffold mutations in 293 T cells. Our system could form the basis for the construction of sophisticated chain reaction systems in living cells.

## Results

### Two strategies to generate cellular chain reactions using nCas9-CDA

**Figure 1:**
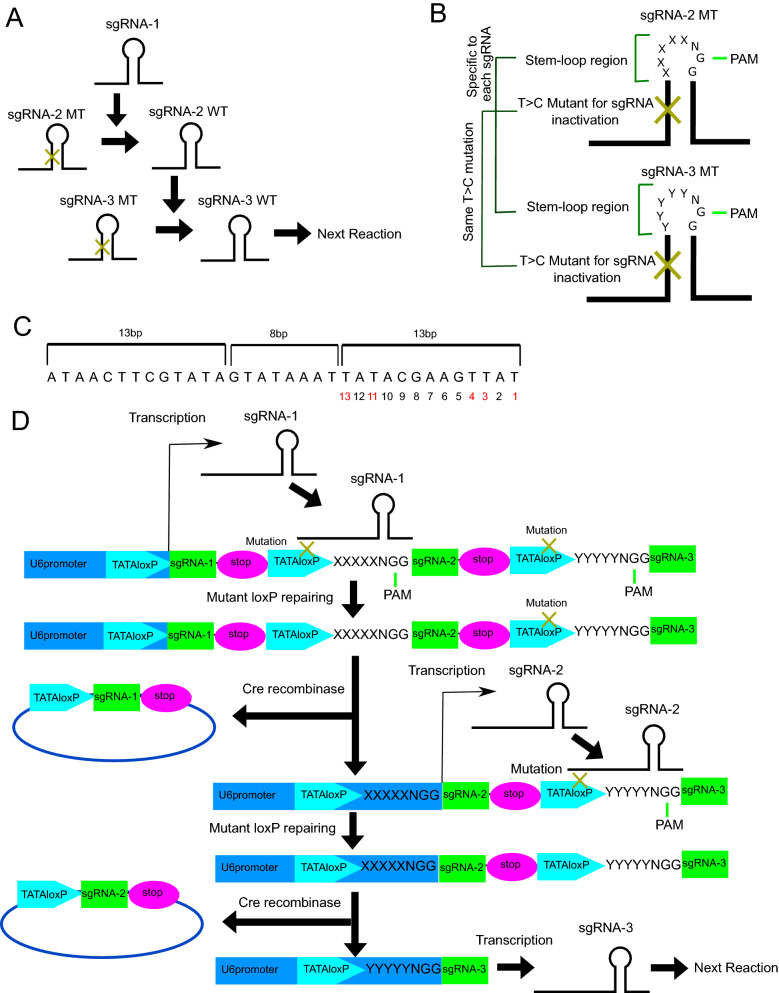
Screening of inactive sgRNAs with scaffold mutations in yeast. (**A**) Schematic representation of the chain reaction with nCas9-CDA and sgRNAs with scaffold mutations. (**B**) Schematic representation of the sgRNA structure with the T > C mutation and specific sequences in the stem-loop region. (**C**) Schematic representation of TATAloxP structure and sequences. (**D**) Schematic representation of chain reaction with nCas9-CDA and TATAloxP with T > C mutations.

We designed two systems to generate chain reactions using nCas9-CDA. In the first system, the T > C mutations are introduced in the scaffold region of sgRNA to disrupt its structure. First sgRNA without mutation (sgRNA-1) and nCas9-CDA correct the T > C mutations in the second sgRNA (sgRNA-2). The corrected sgRNA-2 in turn corrects the T > C mutations in the third sgRNA (sgRNA-3) in cooperation with nCas9-CDA (Fig. 1A). Because the sequence and the length of the stem-loop region in the sgRNA scaffold are not restrictive^4,5^, we can design multiple sgRNAs by introducing various sequences in the stem-loop region (XXXXX in sgRNA-2-MT, YYYYY in sgRNA-3-MT) (Fig. 1B).

In the second system, the T > C mutations are introduced in the TATAloxP sequence (TATAloxP-MT). TATAloxP forms a 13-8-13 structure, which is composed of an 8-bp spacer sequence containing TATA box flanked on either side by two 13-bp inverted repeats (Fig. 1C). Cre recombinase binds to the 13 bp repeats of the TATAloxP, and importantly, this TATA box containing sequence can be incorporated into the U6 promoter to induce sgRNA expression^6^. The T > C mutations in the TATAloxP are corrected by the nCas9-CDA and the sgRNA (sgRNA-1) targeting the mutations and adjacent sequences (XXXXX in Fig. 1D). After the correction, a loxP flanked stop cassette is excised by Cre recombinase, which drives expression of the second sgRNA (sgRNA-2). The sgRNA-2 targets the next mutation and adjacent sequences (YYYYY in Fig. 1D), resulting in expression of the third sgRNA (sgRNA-3) (Fig. 1D). Because there is no restriction for the adjacent sequences, we can design multiple sgRNAs with various adjacent sequences that can be activated during the chain reaction.

### Generation of inactive sgRNAs with T > C mutations in the scaffold region

**Figure 2:**
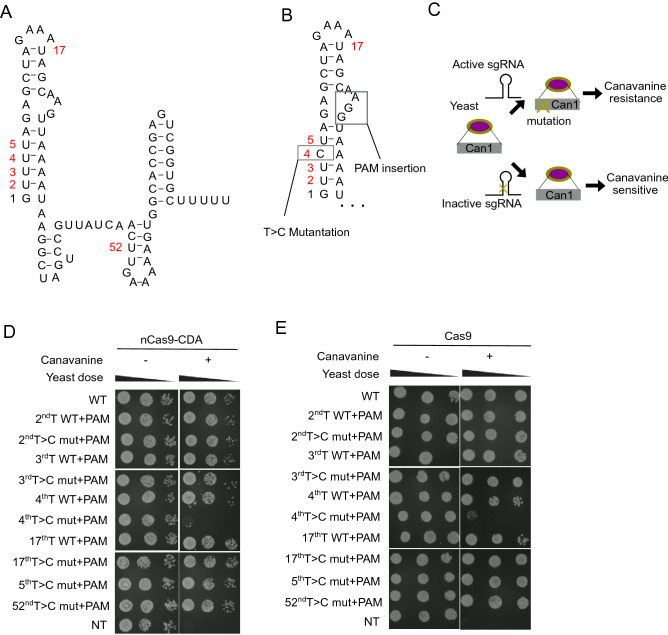
Screening of inactive sgRNAs with scaffold mutations in yeast. (**A**) Schematic representation of the scaffold region of sgRNAs. (**B**) Schematic representation of part of the scaffold region of sgRNAs with T > C mutations and PAM insertion. (**C**–**E**) Schematic representation (**C**) and results of the canavanine assay with nCas9-CDA (**D**) or Cas9 (**E**). Yeasts transduced with the indicated sgRNA were grown on plates with (right column) or without (left column) canavanine.

To establish the first system, we introduced T > C mutations into the template sequence of the sgRNA scaffold region to inactivate it. Because nCas9-CDA was shown to efficiently induce C > T substitution in DNA when the cytosine was located 18 bp upstream of PAM sequence^3^, we also introduced PAM sequence (NGG) 18 bp downstream of the T > C mutation in each sgRNA template (Fig. 2A,B). We then evaluated the activity of each sgRNA with the T > C mutations and PAM insertion using the canavanine assay. Canavanine is a toxic analog of arginine and is imported into yeast cells via a transporter Can1. Therefore, expression of Cas9 and Can1-targeting sgRNA results in depletion of Can1 and decreases the sensitivity of yeast to canavanine (Fig. 2C)^7^. We used this assay to evaluate the effects of each T > C mutation on sgRNA inactivation and found that the T > C mutation at the fourth base resulted in sgRNA inactivation in yeast. Insertion of the PAM sequence did not inhibit sgRNA function in yeast (Fig. 2D,E).

**Figure 3:**
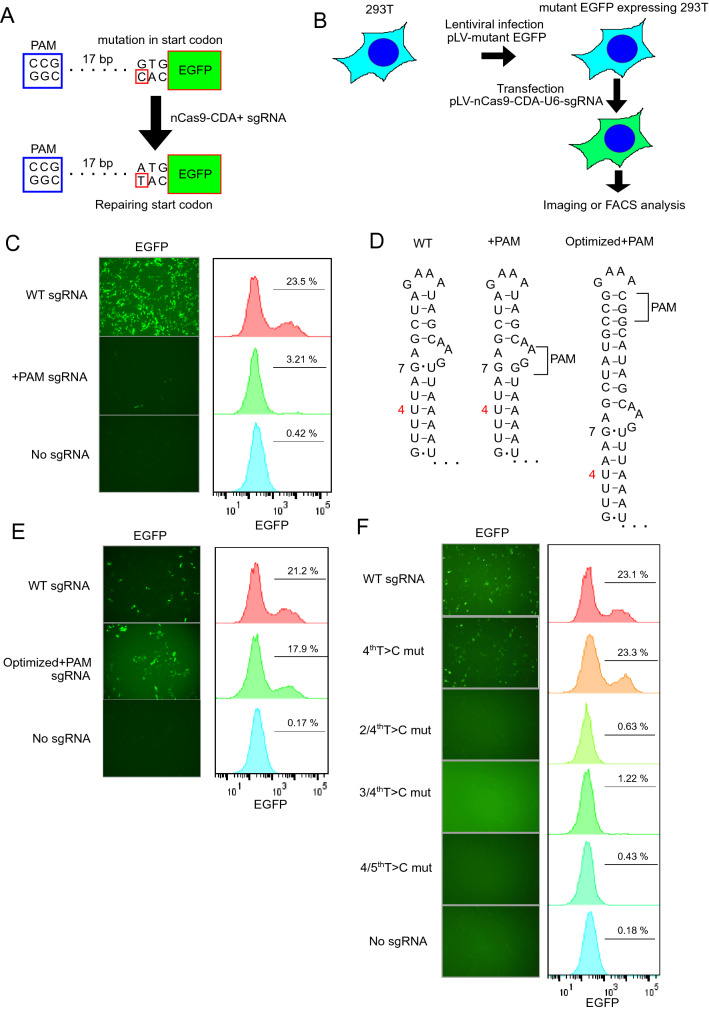
Screening of inactive sgRNAs with scaffold mutations in mammalian cells. (**A**) Schematic representation of wild-type and mutant (ATG to GTG) EGFP with PAM sequence insertion. (**B**) Experimental scheme used in (**C**,**E**–**F**) and Fig. 4A. 293 T cells were transduced with the EGFP mutant. The mutant EGFP-expressing 293 T cells were then transfected with nCas9-CDA and sgRNAs targeting the T > C mutation. (**C**) Fluorescence images (left) and FACS plots (right) of 293 T cells expressing mutant EGFP and nCas9-CDA together with wild-type (top), PAM-inserted (middle), or no (bottom) sgRNA. (**D**) Schematic representation of wild-type (left) and PAM-inserted sgRNAs (original version: middle, optimized version: right). Note that the G-U interaction at the 7th base is lost in the original PAM-inserted sgRNA. The optimized version of the PAM-inserted sgRNA contains additional sequences in the stem-loop region to preserve the G-U interaction at the seventh base. (**E**) Fluorescence images (left) and FACS plots (right) of 293 T cells expressing mutant EGFP and nCas9-CDA together with wild-type (top), PAM-inserted (middle), or no (bottom) sgRNA. (**F**) Fluorescence images (left) and FACS plots (right) of 293 T cells expressing mutant EGFP and nCas9-CDA together with different sgRNAs. The indicated T > C mutations were introduced into the control sgRNA. mut; mutation.

**Figure 4:**
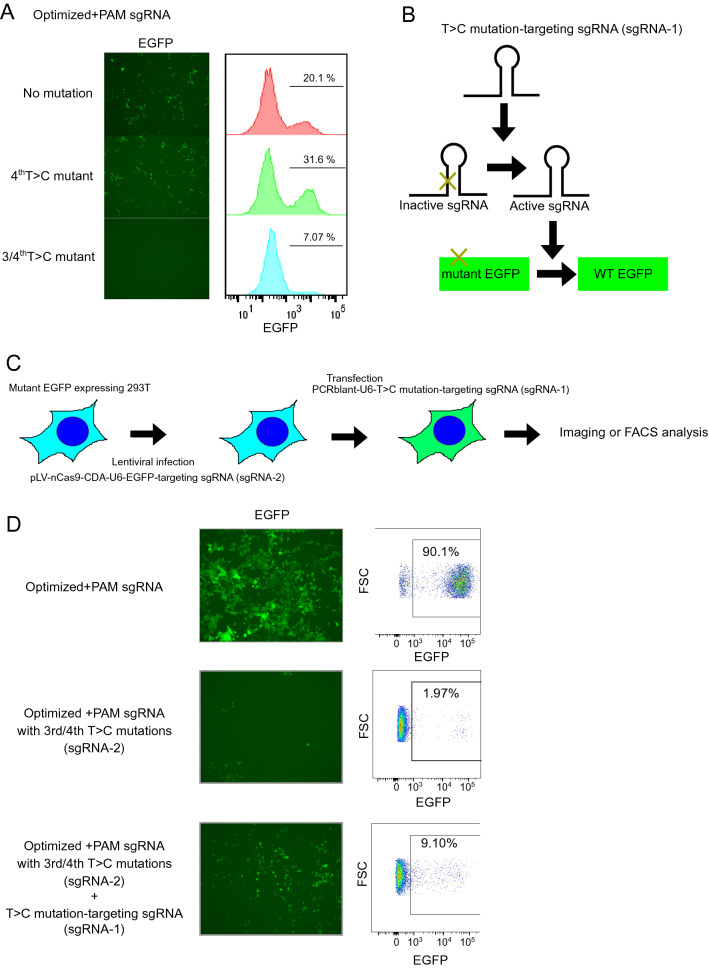
Cellular chain reaction systems through sequential repair of sgRNA scaffold mutations. (**A**) Fluorescence images (left) and FACS plots (right) of 293 T cells expressing mutant EGFP and nCas9-CDA together with different sgRNAs. The indicated T > C mutations were introduced into the optimized PAM-inserted sgRNA. mut; mutation. (**B**) Schematic representation of the chain reaction by repair of sgRNA scaffold mutations to express EGFP in 293 T cells. (**C**) Experimental scheme as in (**D**). 293 T cells were transduced with mutant EGFP, nCas9-CDA and an EGFP-targeting sgRNA, then transfected with another sgRNA targeting the T > C mutation. (**D**) Fluorescence images (left) and FACS plots (right) of 293 T cells expressing mutant EGFP and nCas9-CDA together with the indicated sgRNAs. The sgRNA-1 converted the inactive sgRNA-2 to an active form, and the sgRNA-2 corrected the EGFP mutation to induce EGFP expression.

We then examined the effects of T > C mutations on sgRNA function in mammalian cells. An EGFP mutant was used in which the start codon was mutated from ATG to GTG^8^. In this EGFP mutant, the template strand has a T > C mutation at the start codon and a PAM sequence at its 18 bp downstream. Therefore, nCas9-CDA and active sgRNA can correct the T > C mutation in the EGFP mutant and induce EGFP expression (Fig. 3A,B). However, the sgRNA did not work when a PAM sequence was inserted into the scaffold region of sgRNA, in contrast to the results of the canavanine assay (Fig. 3C). Because the weak interaction between G and U is known to be important for the maintenance of RNA structure^9^, we suspected that the loss of G-U interaction at the seventh base caused by PAM insertion might affect sgRNA function (Fig. 3D). Therefore, we introduced additional sequences near the PAM sequence to maintain the G-U interaction at the 7th base, because extending the stem-loop sequences in the sgRNA scaffold does not affect sgRNA function^4,5^. As expected, this optimized PAM-inserted sgRNA maintained the G-U interaction at the 7th base (Fig. 3D) and efficiently induced EGFP expression when expressed with nCas9-CDA and mutant EGFP in 293 T cells (Fig. 3E). We next examined whether the T > C mutation at the 4th base, which decreased sgRNA activity in yeast, also inactivated sgRNA in mammalian cells. Again, we obtained different results in mammalian cells from those in the canavanine assay. The sgRNA with a single T > C mutation at the fourth base did not inactivate GFP expression, indicating that it retained its normal function. Therefore, in addition to the 4th base, we introduced T > C mutations at 2, 3, or 5 bases into the sgRNA template. These sgRNAs with double T > C mutations at 2/4th, 3/4th, 4/5th bases lost the function to restore GFP expression in 293 T cells (Fig. 3F). We then performed similar experiments with the PAM-inserted sgRNAs and confirmed that the double T > C mutations at 3/4 bases, but not the single T > C mutation at the 4th base, resulted in loss of sgRNA function (Fig. 4A). Thus, we generated an inactive sgRNA with T > C mutations and PAM sequence in the scaffold region whose function can theoretically be restored by nCas9-CDA-induced base editing in mammalian cells.

### Establishment of a chain reaction by repair of sgRNA scaffold mutations

Next, we investigated whether the inactive sgRNA with T > C mutations can be converted to the active form by nCas9-CDA-induced base editing in mammalian cells. First, we transduced nCas9-CDA, the EGFP mutant, a PAM-inserted control or inactive sgRNA targeting the EGFP mutation, with or without the second sgRNA in 293 T cells. The second sgRNA was designed to correct the T > C mutations in the EGFP-targeting sgRNA and restore it to an active form (Fig. 4B,C). As expected, the optimized version of the PAM-inserted sgRNA efficiently corrected the EGFP mutation and induced GFP expression, whereas the version with T > C mutation did not. Importantly, coexpression of the second sgRNA restored the function of the inactive sgRNA and resulted in robust GFP expression in 293 T cells (Fig. 4D). Thus, the chain reaction was successfully established in mammalian cells with nCas9-CDA and two sgRNAs targeting the T > C mutation or EGFP mutation.

### Generation of non-responsive TATAloxP sequences with T > C mutations

**Figure 5:**
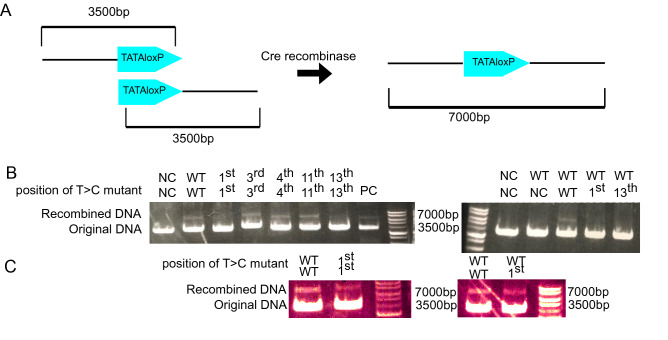
In vitro Screening of non-responsive TATAloxP with T > C mutations. (**A**) Schematic representation of the in vitro recombination assay with TATAloxP sequences. (**B**,**C**) Linear DNAs with different TATAloxP sequences containing the indicated mutations were incubated with Cre recombinase. Note that TATAloxP with the T > C mutation at the first base probably did not efficiently produce recombined DNA in the first experiment because of technical errors (**B**), but did so very well in the second experiment (**C**). (NC; No loxP, PC; positive control DNA in Cre recombinase (NEB Catlog# M0298S)).

To establish the second system, we introduced T > C mutations into the TATAloxP sequences to make them insensitive to Cre-induced recombination. We generated several TATAloxP mutants in which a T base in the 13-bp repeat sequence (Fig. 1C) was replaced by C. These DNAs with different TATAloxP sequences at the 5′ or 3′ side (approximately 3500 bp-vector) were linearized and incubated with Cre recombinase. In this in vitro assay, Cre-induced recombination results in linear DNA of approximately 7000 bp when Cre recombinase can recognize the TATAloxP sites (Fig. 5A). As shown in Fig. 5B,C, the T > C mutation at the 13th base resulted in reduced recombination in both mutant/mutant and mutant/wild-type incubations.

**Figure 6:**
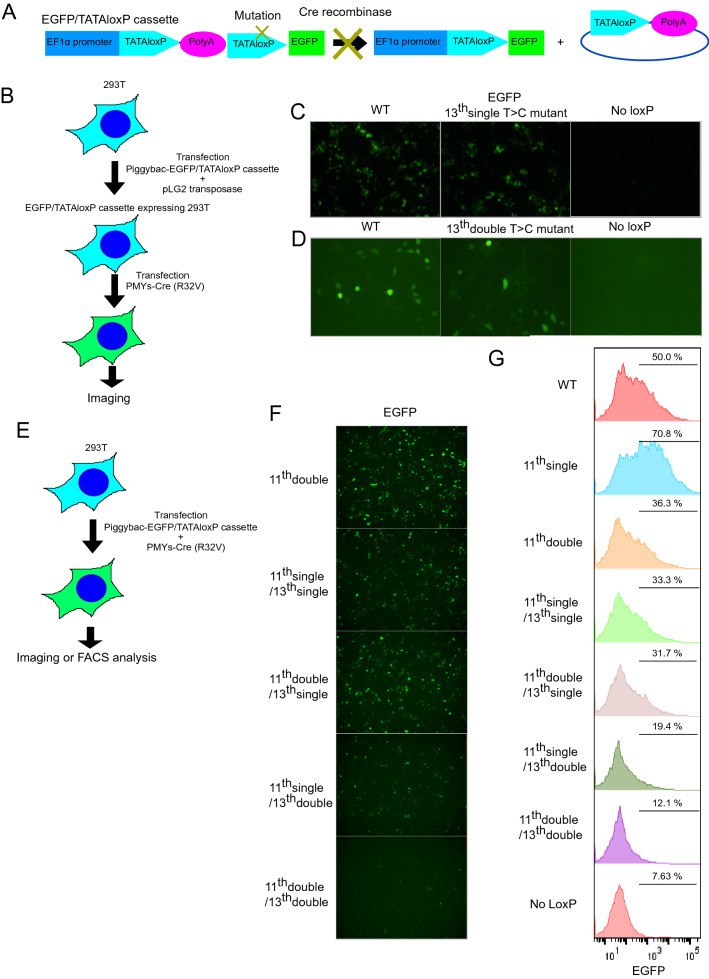
Screening of non-responsive TATAloxP with T > C mutations in mammalian cells. (**A**) Schematic representation of the EGFP cassette with mutated TATAloxP and polyA sequences under the EF1α promoter. (**B**) Experimental scheme used in (**C**,**D**). 293 T cells were transduced with the EGFP/TATAloxP cassette, and the mutant EGFP-expressing 293 T cells were subsequently transfected with Cre-R32V. (**C**,**D**) Fluorescence images of 293 T cells expressing EGFP/ various TATAloxP with the indicated T > C mutations and Cre-R32V. (**E**) Experimental scheme as in (**F**,**G**). 293 T cells were transfected with EGFP/TATAloxP cassette and Cre-R32V. (**F**,**G**) Fluorescence images (**F**) and FACS plots (**G**) of 293 T cells expressing EGFP/various TATAloxP with the indicated T > C mutations and Cre-R32V. WT; wild type.

We next investigated whether the TATAloxP sequences containing the T > C mutations are resistant to Cre-mediated recombination in mammalian cells. We generated an expression vector containing a polyA signal flanked by two TATAloxP sites and a downstream EGFP cassette under the EF1α promoter in which one of the TATAloxP sites has the T > C mutations. If the mutated TATAloxP site is resistant to Cre-mediated recombination, EGFP is not expressed even in the presence of Cre recombinase (Fig. 6A). We expressed the TATAloxP (wild-type or mutant)-EGFP constructs in 293 T cells together with Cre-R32V, a mutant Cre recombinase with improved fidelity^10^ (Fig. 6B). However, in contrast to the results of the in vitro experiments, the T > C mutation at 13 base did not prevent Cre-R32V-induced EGFP expression in either or both 13-bp arms (Fig. 6C,D). Thus, the T > C mutation at the 13th base was not sufficient to render the TATAloxP sequence insensitive to Cre recombinase in mammalian cells. Because a previous report showed that double mutations in both 13-bp arms, particularly at the 7th, 8th, 11th, 12th, and 13th bases efficiently disrupted the loxP structure^11^, we subsequently examined the effect of double T > C mutations at the 11th and/or 13th bases on their responsiveness to Cre recombinase. Among the different combinations, we found that double T > C mutations at the 11th and 13th bases became resistant to Cre-induced EGFP expression in both arms (Fig. 6E,F,G). In this way, we generated a TATAloxP mutant which does not respond to Cre but will resume the responsiveness to Create by nCAS9-CDA-induced base editing in mammalian cells.

### Inefficient repair of TATAloxP mutations in the chain reaction system

**Figure 7:**
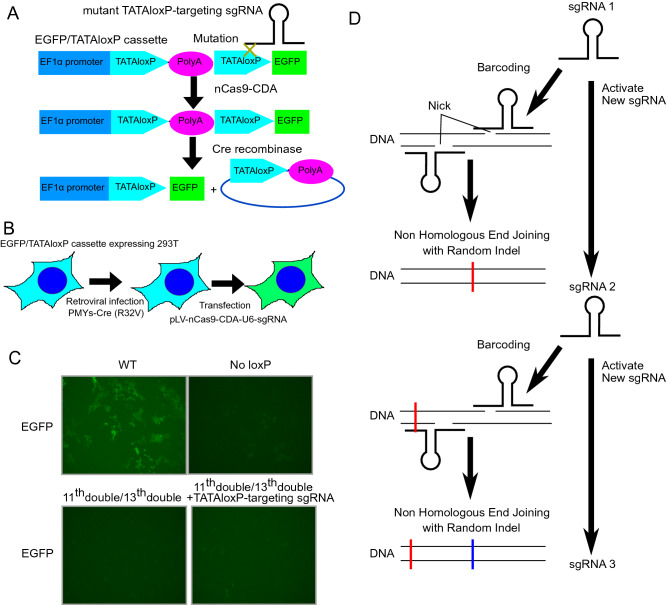
Cellular chain reaction systems through sequential repair of loxP mutations. (**A**) Schematic representation of the chain reaction by repair of TATAloxP mutations to express EGFP in 293 T cells. (**B**) Experimental scheme used in (**C**). 293 T cells were transduced with the EGFP/different TATAloxP cassette with the indicated T > C mutations, then infected with Cre-R32V, and then transfected with nCas9-CDA and sgRNA targeting the T > C mutation. (**C**) Fluorescence images of 293 T cells expressing EGFP/TATAloxP cassette, Cre-R32V, and nCas9-CDA along with the indicated sgRNAs. When the EGFP/TATAloxP cassette was used with T > C mutations at the 11th and 13th bases in both arms, no EGFP expression was detected, even in the presence of the TATAloxP-targeting sgRNA (bottom right). (**D**) Schematic representation of barcoding method.

Finally, we examined whether we could establish the cellular chain reaction system using the TATAloxp mutant. We transduced wild-type and nonresponsive TATAloxP-EGFP, Cre-R32V, and nCas9-CDA into 293 T cells with or without the second sgRNA. The second sgRNA was designed to correct the T > C mutations in the nonresponsive TATAloxP and convert it to a responsive form (Fig. 7A,B). Consistent with previous results, Cre-R32V induced recombination of only the wild-type TATAloxP but not the mutant TATAloxP to induce EGFP expression in 293 T cells (Fig. 7C). Unfortunately, coexpression of the second sgRNA failed to restore EGFP expression, indicating that the TATAloxP mutant with double T > C mutations at bases 11 and 13 is resistant to nCas9 DNA-mediated base editing. Thus, this TATAloxP system in its current form is not suitable for cellular chain reactions.

## Discussion

To develop a system that enables sequential sgRNA activation in mammalian cells, we designed two chain reaction systems using the nCas9 CDA base editor. As a proof of concept, we successfully established a chain reaction by repairing sgRNA scaffold mutations in 293 T cells. In our system, there is no theoretical limit to the number of sequential reactions. Moreover, our system allows the activation of different sgRNAs at each stage of the chain reaction. Thus, our chain reaction system provides a useful platform for recording and controlling molecular events in living cells. For example, in combination with the cell cycle-dependent CRISPR-Cas9 activation system^12^, our chain reaction system can be used to develop a counter for cell division. Previously, several methods to monitor cell division have been reported. Those using BrdU or H2B-EGFP dilution were used to distinguish cells that divided more than 4–5 times from those divided less^13,14^. Another method using Ki67-Cre was used to mark cells that divided at least once^15^. In contrast to these methods that only record a few cell divisions, our system has the potential to serially count each cell division. With such systems, we will be able to know how many times a stem cell divides to be a terminally differentiated cell. In addition, it will be interesting to combine the chain reaction system with the DNA barcoding technique. It is known that nCas9 can cause random indel by adding a nick in two places^16^. This random indel can be used as a “barcode”. Therefore, combined with the cell division counting system described above, it is theoretically possible to introduce a barcode to each daughter cell at each cell division (Fig. 7D). Such systems will enable us to draw the exact cell lineage trees during tissue development, which is impossible with the current DNA barcoding systems.

However, further optimization will be required to develop more sophisticated chain reaction systems in mammalian cells. Although we achieved the chain reaction using the sgRNA scaffold mutation system, the activity of the repaired sgRNA was much weaker than that of the control sgRNA. We could not even restore the reactivity of mutant TATAloxP by nCas9-CDA-mediated base editing in mammalian cells. To achieve more robust chain reactions, it is necessary to increase the mutation repair rate at each step. In order to improve the repair efficiency of mutant sgRNAs, further screening is required for finding mutants that lose their function after only a single mutation or adjusting the sequence of the stem loop region of sgRNAs which is changeable without loss of function with checking repairing rate. Our study also showed that the results obtained in yeast or in vitro are not the same as those obtained in mammalian cells. It has been shown that physiological RNA structure depends not only on sequence but also on RNA modifications and binding proteins^17^. DNA methylation has also been shown to affect the efficiency of Cre-mediated recombination^18^. These in vivo-specific regulatory mechanisms could explain the discrepancy between the in vitro and in vivo results. Thus, our results strongly suggest that in vivo chain reactions need to be optimized in appropriate cellular contexts.

In summary, we have developed a cellular chain reaction system using nCas9 CDA-mediated base editing of sgRNA scaffold mutations. Our system, in combination with tissue-specific or time-dependent regulation, may have broad utility for future biomedical research.

## Methods

### Plasmids

Plasmids and primers used for plasmid construction are summarized in supplemental Table 1. Mutations were introduced by PCR with KOD FX NEO (TOYOBO), and mutants were cloned into vectors with Gibson assembly using Gibson Assembly Master Mix (NEB). The TATAloxP sequence was generated using gBlocks (IDT). For plasmid construction of pNMA001, the gRNA coding region was constructed by PCR with primers (5′-GTTTTAGAGCTAGAAATAGCAAGTTAAAATAAGGCTAGTCCGTTATCAACTTGAAAAAGTGGCACCGAGTCGGTGCTTTTTTGTTCACTGCCGTATATAGGCAG-3′ and 5′-AAACTTCTCCGCAGTGAAAGATAAATGATCGTCAATTACGAAGACTGAACGTTTTAGAGCTAGAAATAGC-3′). The amplified product and the p426-SNR52p-gRNA.CAN1. Y-SUP4t (Addgene#43,803) backbone fragment amplified by PCR using primers (5′- GATCATTTATCTTTCACTGCGG-3′ and 5′-TGTTTTTATGTCTTCGAGTCATGTAATTA-3′) were subsequently assembled by Gibson Assembly. For plasmid construction of pLV-U6p-gRNA-CMVp-nCas9-PmCDA1-UGI-2A-mCherry, the human U6 promoter and gRNA coding region were amplified by PCR from plasmid pSI-236 using primers (5′-GGGACAGCAGAGATCCAGTTATCGATGAGGGCCTATTTCCCATG-3′) and (5′-GTAATTGATTACTATTAATAACTAGTAAAAAAGCACCGACTC GGT-3′). The amplified product and the pLV-CS-086 backbone fragment digested with SpeI and ClaI were subsequently assembled using Gibson Assembly.

### Cell culture

293 T cells (CRL-11268, ATCC, Manassas, VA, USA) were cultured in DMEM (Wako) containing 10% fetal bovine serum (FBS) (Biowest) at 37 °C and 5% CO2. 293 T was authenticated by short tandem repeat analyses and tested for mycoplasma contamination in our laboratory. For selection, 1 μg/ml puromycin (Thermo Fisher Scientific), 5 μg/ml blasticidin S (Thermo Fisher Scientific), and 1 mg/ml zeosin (InvivoGen) were used.

### Transient transfection

Plasmids were transfected using the calcium phosphate co-precipitation method^19^. A mixture of 10–20 μg plasmid (10–20 μl), 2.5 M CaCl2 (50 μl), and filtered water (430–440 μl) to 2 × HeBS (500 μl) was added to 293 T cells in a 10-cm dish (2.0 × 10^6^ cells/dish) for 18 h. To integrate the piggyback vector, 15 μg of piggyback vector and 5 μg of pLG2 (PB200PA-1, SBI) were co-transfected with the above plasmids.

### Viral transduction

Lentiviruses and retroviruses were prepared by transient transfection in 293 T cells using the calcium phosphate method as described above. A mixture of plasmids (3 μg VSVG and 10 μg PAX2 for lentiviruses, 3 μg RD114 and 10 μg M57 for retroviruses along with 12 μg viral vectors), 2.5 M CaCl2 (50 μl), and filtered water (425 μl) to 2 × HeBS (500 μl) was added to 293 T cells in a 10-cm dish (2.0 × 10^6^ cells/dish). After 24 h, the medium was removed by aspiration and 10 ml of fresh DMEM(Wako)/10% FBS (biowest) was added. The virus-containing medium was collected 48 h after transfection.

### In vitro Cre recombinase reaction assay

One μg of the mutant TATAloxP sequences in the zero-blant vector (Thermo Fisher Scientific) were incubated with 1.5 U/ml BamHI, 1.0 U/ml NotI or 1.0 U/ml MluI to linearize them. The linear DNAs (20 ng each) were mixed with 10 × Cre Recombinase Reaction Buffer, Cre Recombinase and H2O and incubated at 37 °C for 30 min and then at 70 °C for 10 min.

### Live cell imaging

Live cell imaging was performed using the Invitrogen EVOS FL Auto 2 Imaging System (Thermo Fisher Scientific). Phase contrast images were acquired using a 10 × phase contrast objective and camera (with a resolution of 1388 × 1040 pixels). EGFP images were acquired using the EVOS™ LED cube, GFP.

### Flowcytometry analysis

The 293 T cells were resuspended in PBS containing 2% FBS. Cells were then analyzed using a FACS-Verse (BD Biosciences).

### Canavanine assay

Saccharomyces cerevisiae strain BY4741 (MATa his3Δ0leu2Δ0 met15Δ0 ura3Δ0) was used for CAN1 mutagenesis analysis of the CRISPR system. Parent strains BY4741 were grown before transformation in YPAD and then propagated in the appropriate synthetic complete media (SC) without the auxotrophic compound supplemented with the plasmids. BY4741 were spread onto the YPAD plate on day 1. On day 3, BY4741 single colonies were harvested and cultured in 5 ml YPAD at 30℃ overnight. On day 4, transformation of plasmids containing a galactose-inducible modifier gene plasmid (Cas9, nCas9-CDA) with LEU2 marker and a gRNA-expressing plasmid with Ura3 marker (250 μg each per transformation) was performed using a Frozen EZ Yeast Transformation Kit (ZYMO RESEARCH). After transformation, BY4741 were spread on SC-Leu-Ura + Ade plates for selection. On day 6, single colonies were taken from each plate and cultured in 5 ml SC-Leu-Ura-Ade at 30℃. On day 8, the culture medium was changed to SRaffi-Leu-Ura + Ade. On the 10th day, the culture medium was replaced with SGall-Leu-Ura + Ade. On day 11, BY4741 were spread on SC-Leu-Ura + Ade + canavanine plates or SC-Leu-Ura + Ade plates.

## Supplementary Information

Supplementary Information.

## Data Availability

Information and inquiries about reagents can be directed to the main contact person, Yosuke Tanaka (ytims@ims.u-tokyo.ac.jp).
