# Supplementary material for: CRISPR/Cas9-mediated base-editing enables a chain reaction through sequential repair of sgRNA scaffold mutations
Source: Sci Rep. 2021 Dec 13;11:23889. doi: 10.1038/s41598-021-02986-6 (PMC8668876; doi:10.1038/s41598-021-02986-6)
Supplement: Supplementary file 1 — Supplementary Information. [file 41598_2021_2986_MOESM1_ESM.pdf]

Supplementary figures and tables for

CRISPR/Cas9-mediated base-editing enables a chain reaction through sequential repair of sgRNA scaffold mutations

Tsuyoshi Fukushima, Yosuke Tanaka, Keito Adachi, Nanami Masuyama, Akiho Tsuchiya, Shuhei Asada, Soh Ishiguro, Hideto Mori, Motoaki Seki, Nozomu Yachie, Susumu Goyama, Toshio Kitamura

FigureS1

Full blot of Figure 5B

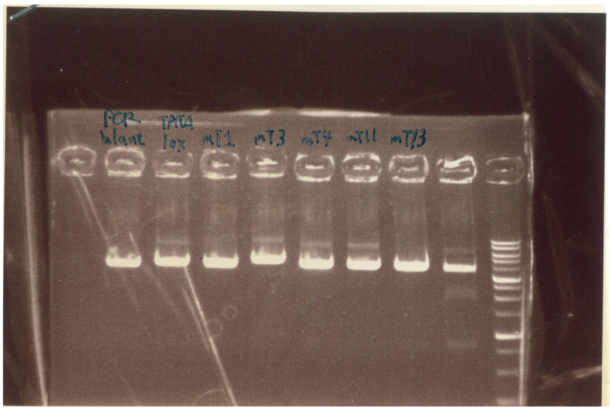

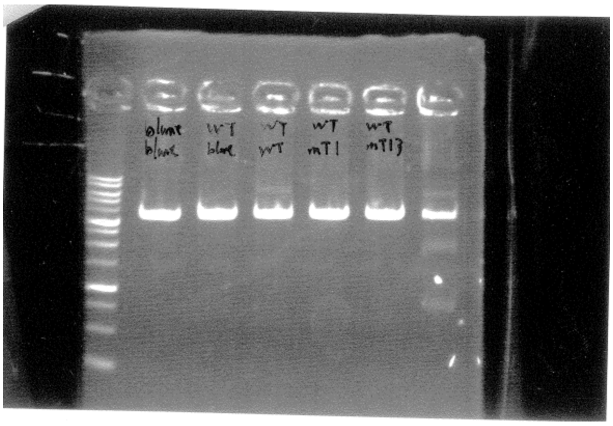

Full blot of Figure 5C

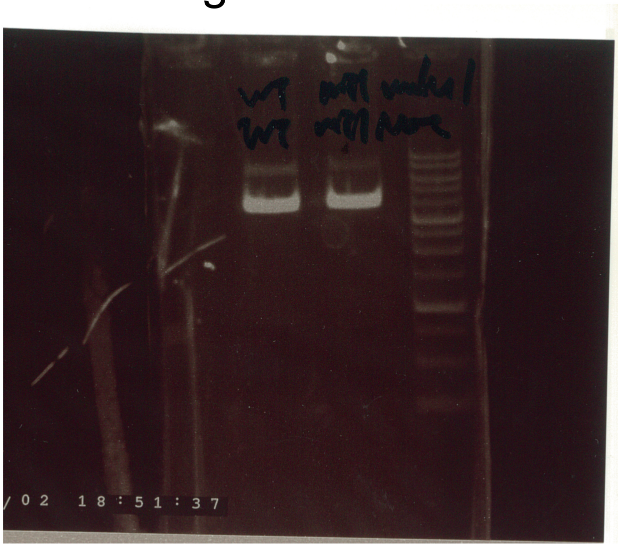

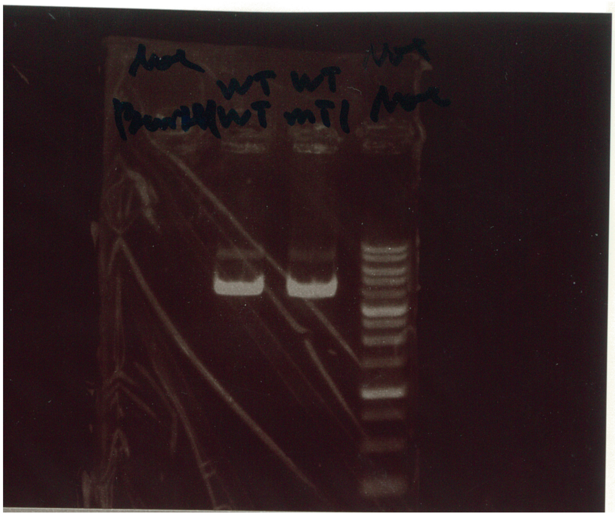

Table S1: Plasmid

Vector	Insert	Reference
pKN1252	nCas9-PmCDA1-ugi-LEU2 marker	Nisida et al[3]
pKN1252	Cas9-LEU2 marker	Nisida et al[3]
pKN1085	Can1-targeting sgRNA-Ura3 marker	Nisida et al[3]
pNMA001	ADE1-targeting sgRNA-Ura3 marker	In this study
pKN1085	Can1-targeting sgRNA (with 2nd T>C mutation+PAM) containing Ura3 marker	In this study
pKN1085	Can1-targeting sgRNA (with PAM for 2nd T>C mutation) containing Ura3 marker	In this study
pKN1085	Can1-targeting sgRNA (with 3rd T>C mutation+PAM) containing Ura3 marker	In this study
pKN1085	Can1-targeting sgRNA (with PAM for 3rd T>C mutation) containing Ura3 marker	In this study
pKN1085	Can1-targeting sgRNA (with 4th T> mutation +PAM) containing Ura3 marker	In this study
pKN1085	Can1-targeting sgRNA (with PAM for 4th T>C mutation)-Ura3 marker	In this study
pKN1085	Can1-targeting sgRNA (with 17th T>C mutation+PAM)-Ura3 marker	In this study
pKN1085	Can1-targeting sgRNA (with PAM for 17th T>C mutation)-Ura3 marker	In this study
pKN1085	Can1-targeting sgRNA (with 5th T>C mutation+PAM)-Ura3 marker	In this study
pKN1085	Can1-targeting sgRNA (with 52rd T>C mutation+PAM)-Ura3 marker	In this study
pLV	CMVp-nCas9-PmCDA1-ugi-hU6 promoter-EGFP-targeting-sgRNA-PGK-mCherry	In this study
pLV	CMVp-nCas9-PmCDA1-ugi-hU6 promoter-EGFP-targeting-sgRNA-PGK-bleomycin	In this study
pLV	CMVp-nCas9-PmCDA1-ugi-PGK-bleomycin	In this study
pLV	CMVp-nCas9-PmCDA1-ugi-hU6 promoter- EGFP-targeting-sgRNA (with PAM)-PGK-bleomycin	In this study

pLV	CMVp-nCas9-PmCDA1-ugi-hU6 promoter- EGFP-targeting-sgRNA (with 4th T>C mutation)-PGK-bleomycin	In this study
pLV	CMVp-nCas9-PmCDA1-ugi-hU6 promoter- EGFP-targeting-sgRNA (with 2nd/4th T>C mutation)-PGK-bleomycin	In this study
pLV	CMVp-nCas9-PmCDA1-ugi-hU6 promoter- EGFP-targeting-sgRNA (with 3rd/4th T>C mutation)-PGK-bleomycin	In this study
pLV	CMVp-nCas9-PmCDA1-ugi-hU6 promoter- EGFP-targeting-sgRNA (with 4th/5th T>C mutation)-PGK-bleomycin	In this study
pLV	CMVp-nCas9-PmCDA1-ugi-hU6 promoter- EGFP-targeting-Optimized sgRNA-PGK-bleomycin	In this study
pLV	CMVp-nCas9-PmCDA1-ugi-hU6 promoter- EGFP-targeting-Optimized sgRNA (with PAM)-PGK-bleomycin	In this study
pLV	CMVp-nCas9-PmCDA1-ugi-hU6 promoter- EGFP-targeting-Optimized sgRNA (with 4th T>C mutation PAM)-PGK-bleomycin	In this study
pLV	CMVp-nCas9-PmCDA1-ugi-hU6 promoter- EGFP-targeting-Optimized sgRNA (with 3rd/4th T>C mutation PAM)-PGK-bleomycin	In this study
pLV	CMVp-mutant EGFP-PGK-puromycin	#131127 (Addgene)[8]
Zeroblant	TATAloxP	In this study
Zeroblant	TATAloxP with 1st T>C mutation	In this study
Zeroblant	TATAloxP with 3rd T>C mutation	In this study
Zeroblant	TATAloxP with 4th T>C mutation	In this study
Zeroblant	TATAloxP with 11th T>C mutation	In this study
Zeroblant	TATAloxP with 13th T>C mutation	In this study
PB	CMVp-MCS-EF1ap-puromycin-EGFP	PB531B1 (SBI)
pLG2	CMVp-Transposase	PB210PA-1 (SBI)
PB	EF1αp-polyA-polyA-puromycin-PGKp-TATAloxP-EGFP	In this study
PB	EF1αp-TATAloxP-polyA-polyA-puromycin-PGKp-TATAloxP-EGFP	In this study
PB	EF1αp-TATAloxP (with 13th single T>C mutation)-polyA-polyA-puromycin-PGKp-TATAloxP-EGFP	In this study

PB	EF1αp-TATAloxP (with 13th double T>C mutation)-polyA-polyA-puromycin-PGKp-TATAloxP-EGFP	In this study
PB	EF1αp-TATAloxP (with 11th single/13th single T>C mutation)-polyA-polyA-puromycin-PGKp-TATAloxP-EGFP	In this study
PB	EF1αp-TATAloxP (with 11th double/13th single T>C mutation)-polyA-polyA-puromycin-PGKp-TATAloxP-EGFP	In this study
PB	EF1αp-TATAloxP (with 11th single/13th double T>C mutation)-polyA-polyA-puromycin-PGKp-TATAloxP-EGFP	In this study
PB	EF1αp-TATAloxP (with 11th double/13th double T>C mutation)-polyA-polyA-puromycin-PGKp-TATAloxP-EGFP	In this study
Zeroblant	hU6p-T>C mutation-targeting sgRNA	In this study
pLV	CMVp-nCas9-PmCDA1-ugi-hU6 promoter-TATAloxP-targeting-sgRNA-PGK-bleomycin	In this study
